# Threonine-Based Stimuli-Responsive Nanoparticles with Aggregation-Induced Emission-Type Fixed Cores for Detection of Amines in Aqueous Solutions

**DOI:** 10.3390/polym14071362

**Published:** 2022-03-27

**Authors:** Keita Kataoka, Kazuhiro Nakabayashi, Chen-Tsyr Lo, Hideharu Mori

**Affiliations:** 1 Graduate School of Organic Materials Science, Yamagata University, 4-3-16 Jonan, Yonezawa 992-8510, Japan; wnkktok94@gmail.com (K.K.); nkbysh@gmail.com (K.N.); lochentsyr@mail.nsysu.edu.tw (C.-T.L.); 2 Department of Materials and Optoelectronic Science, National Sun Yat-Sen University, 70 Lienhai Road, Kaohsiung 80424, Taiwan

**Keywords:** core-cross-linked nanoparticles, fluorescent amine detection, aggregation-induced emission, pH-responsive nanoparticles, self-assembled block copolymer

## Abstract

Stimuli-responsive polymeric nanoparticles (NPs) exhibit reversible changes in the dispersion or aggregation state in response to external stimuli. In this context, we designed and synthesized core-shell NPs with threonine-containing weak polyelectrolyte shells and fluorescent cross-linked cores, which are applicable for the detection of pH changes and amine compounds in aqueous solution. Stable and uniform NP(dTh) and NP(Fl), consisting of fluorescent symmetric diphenyl dithiophene (dTh) and diphenyl fluorene (Fl) cross-linked cores, were prepared by site-selective Suzuki coupling reactions in self-assembled block copolymer. NP(Fl) with the Fl unit in the core showed a high fluorescence intensity in different solvents, which is regarded as an aggregation-induced emission-type NP showing strong emission in aggregated states in the cross-linked core. Unimodal NPs were observed in water at different pH values, and the diameter of NP(Fl) changed from 122 (pH = 2) to 220 nm (pH = 11). Furthermore, pH-dependent changes of the fluorescence peak positions and intensities were detected, which may be due to the core aggregation derived from the deprotonation of the threonine-based shell fragment. Specific interactions between the threonine-based shell of NP(Fl) and amine compounds (triethylamine and *p*-phenylenediamine) resulted in fluorescence quenching, suggesting the feasibility of fluorescent amine detection.

## 1. Introduction

Polymeric nanoparticles (NPs), composed of chromophores and conjugated polymers, are of great technological interest because of their unique properties and potential applications [1,2,3,4,5]. Various synthetic strategies have been exploited to manipulate their sizes, topologies, and interfaces, including the chemical structures of the fluorescent and conjugated units, which are generally located in the cores [1,6]. All organic-based designs of polymeric NPs with different combinations of the precisely controlled core and shell structures enable the realization of a wide range of applications, such as optoelectronics, [7,8,9] biological imaging, and sensing [5,10,11,12,13,14,15]. For example, colloidal NPs with densely packed hydrophobic conjugated polymers in their cores, also known as semiconducting polymer dots, have been extensively studied for bioimaging owing to their characteristic far-red/near-infrared emission [1,5,6,16,17,18]. The prerequisites for biological imaging and sensing applications include excellent water solubility and/or water dispersibility as well as high sensitivity for targeted stimuli and molecules in aqueous solutions, which can be determined by the chemical structure and nature of the outermost surface of the polymeric NPs. High fluorescence properties and substantial changes in the fluorescence intensity and/or peak position are also crucial for sensing, which can be governed by the molecular design and environment of the chromophore/conjugated polymer core. Stimuli-responsive fluorescent NPs, which can cause aggregation and dispersion states in response to external stimuli, such as pH, salt, temperature, and interactions with bio-related molecules, have also been established as an efficient method for optical sensing and imaging [19,20,21,22,23]. In addition, polymer-based nanostructured materials have emerged as powerful tools in the sensing fields. For instance, molecularly imprinted polymers have been extensively applied as promising recognition elements for the detection of biomedical makers and pathogens [24]. Another intriguing example involves bioinspired multifunctional plasmonic nanoplatforms with hierarchical structures and thermoresponsive and optical properties, which are applicable for glucose-sensing [25]. Despite the recent remarkable progress in this field, producing more efficient smart polymeric NPs with reasonable water solubility and dispersibility, strong fluorescence, long-term photostability, well-regulated size and morphology, and specific binding for targeted molecules remains a challenge, and requires a rational molecular design with an efficient synthetic approach.

Fluorescent chemical sensors have steadily received more attention in the field of chemical sensors owing to several advantages, such as rapid and conventional detection and high sensitivity [26,27,28]. The detection of amine compounds is of significant interest because they are widely utilized in several applications, such as pharmaceuticals, fertilizers, surfactants, and colorants, and are among the major pollutants detected in landfills, manufacturing sites, soils, and aqueous environments. Various fluorescence systems have been developed for the detection of aliphatic and aromatic amines, such as those using fluorescence chemodosimeters [29], metal–organic frameworks [30], Schiff base complexes [31], metalloporphyrins [32], conjugated polymers [33], and dyes with aggregation-induced emission [34]. Among the various amine compounds, intense efforts have been devoted to the detection of *p*-phenylenediamine (PPD), which is an important intermediate for hair dyes and polymers and has detrimental effects on the environment and human health. Some of the fluorescent approaches that have been developed to detect PPD include fluorescent sensor block copolymers [35], luminol diazotization [36], and fluorene-containing conjugated polymers with pendant carboxylic acid groups [37]. However, because many detector compounds are hydrophobic and possess rigid conjugated structures, a water-soluble fluorescent sensor that demonstrates stable fluorescence in aqueous solutions is required.

**Scheme 1:**
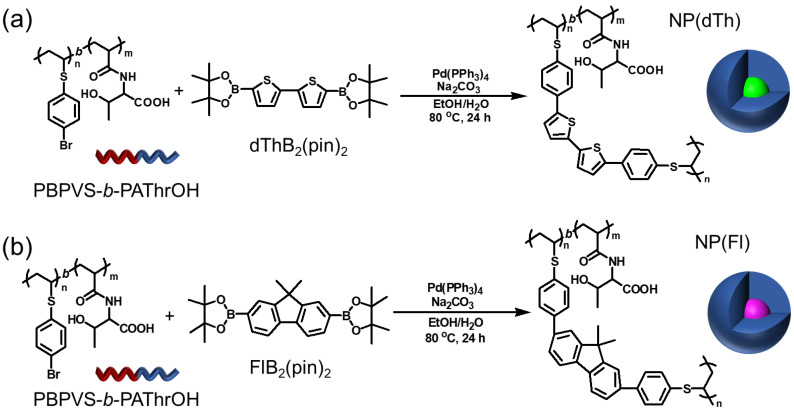
Synthesis of the core-cross-linked fluorescent NPs with a threonine-based polyelectrolyte shell obtained via a one-pot coupling reaction: (**a**) NP(dTh) and (**b**) NP(Fl) using dithiophene (dTh)- and fluorene (Fl)-based diboronic acid esters, respectively.

In this study, we demonstrate the design and synthesis of water-soluble stimuli-responsive NPs that are applicable for the detection of pH changes and amine compounds (aliphatic and aromatic derivatives) by introducing a threonine-containing segment in the shell and fluorescent cross-linked cores (Scheme 1). The corresponding products, denoted as NP(dTh) and NP(Fl), consist of a fluorescent symmetric diphenyl dithiophene (dTh) and diphenyl fluorene (Fl) core, respectively, which are covalently linked to the polymer chain via a thioether unit. A water-soluble and pH-responsive shell derived from *N*-acryloyl-L-threonine (AThrOH) was selected because the hydroxy group in the AThrOH monomer unit contributes to the enhanced water solubility of the core-cross-linked NPs, irrespective of environmental changes, such as pH and addition of amine compounds, whereas the carboxylic acid group acts as a pH-responsive unit.

The design and synthesis of core-cross-linked NPs derived from self-assembled block copolymers is one of the most extensively studied topics in the fields of polymer-based nanotechnology and polymeric micelles [38,39,40,41,42,43,44,45,46,47,48,49,50,51,52]. We have previously developed core-cross-linked NPs with various optoelectronic and conjugated cores, such as electron-rich polythiophene and (di)thiophene [47,48,49,53], electron-transporting rylene bisimide [50], fluorescent anthracene [51], π-conjugated dithiophene [49], donor-acceptor [54,55], and tetraphenylethylene-based aggregation-induced emission (AIE) units [56,57]. Following micelle formation in selective solvents, an in situ Suzuki coupling reaction was conducted with diboronic acids, leading to the transformation of reactive block copolymers into stable core-cross-linked NPs with optoelectronic cores [47,48,49,53,54,55,56,57]. In this study, an efficient synthetic method was adopted in which amphiphilic block copolymers, with the hydrophilic segment derived from AThrOH and the cross-linkable hydrophobic segment derived from 4-bromophenyl vinyl sulfide (BPVS), were synthesized by addition-fragmentation chain-transfer (RAFT) polymerization and one-pot core cross-linking of the assembled block copolymers by Suzuki coupling reactions. Distinct from fluorescent block copolymers, in which local concentrations of the fluorescent units are significantly affected by their environments, the core-cross-linked NPs have a fixed fluorescent unit in the cores, leading to long-term photostability, while change in the relative fluorescence intensity can be employed to detect pH changes and the presence of amine compounds. Variations in the optical and sensing properties of the synthesized materials corresponding to pH changes and different amine compounds were evaluated by fluorescence measurements.

## 2. Materials and Methods

### 2.1. Materials

2,2-Azobis(isobutyronitrile) (AIBN, 97%, Kanto Chemical, Tokyo, Japan) was purified by recrystallization from methanol. 4-Bromophenyl vinyl sulfide (BPVS) [58,59], *N*-acryloyl-L-threonine (AThrOH) [60], and benzyl 1-pyrrolecarbodithioate [61,62] were synthesized, as reported previously. 2,7-Bis(4,4,5,5-tetramethyl-1,3,2-dioxaborolan-2-yl)-9,9-dimethylfluorene (FlB_2_(pin)_2_, >98%, TCI, Tokyo, Japan), 2,2′-bithiophene-5,5′-diboronic acid bis(pinacol) ester (dThB_2_(pin)_2_, >97%, Sigma-Aldrich, St. Louis, MO, USA), sodium carbonate (Na_2_CO_3_, >99%, Wako, Osaka, Japan), and tetrakis(triphenylphosphine)palladium(0) (Pd(PPh_3_)_4_, >97%, TCI) were used as received.

### 2.2. Synthesis of Threonine-Containing Amphiphilic Block Copolymer

Initially, PBPVS macro-CTA was synthesized using BPVS as a bromine-containing monomer and benzyl 1-pyrrolecarbodithioate as a chain transfer agent (CTA, Tables S1 and S2 and Figures S1–S3), according to a reported procedure [49]. Amphiphilic block copolymer, PBPVS-*b*-PAThrOH, was synthesized as follows: AThrOH (0.692 g, 4.00 mmol), PBPVS macro-CTA (0.070 g, 0.02 mmol, *M*_n,NMR_ = 3,500, *M*_w_/*M*_n_ = 1.37), AIBN (1.60 mg, 0.01 mmol), and 4.0 mL of DMF were placed in a dry ampule. After the mixture was degassed by three freeze–evacuate–thaw cycles, the ampule was flame-sealed under vacuum, and then it was heated at 80 °C for 24 h with stirring. The reaction mixture was purified by precipitation in a large excess of ethyl acetate, and then isolated by filtration. Finally, the resultant product was dried under vacuum at room temperature to afford PBPVS-*b*-PAThrOH (0.411 g, Yield = 54%, Table S3). The block copolymer was soluble in DMF, DMSO, ethanol, methanol, water, and insoluble in diethyl ether, acetone, *n*-hexane, ethyl acetate, dichloromethane, chloroform, THF.

### 2.3. Synthesis of Fluorescent NPs with a Threonine-Based Polyelectrolyte Shell

In analogy to previously published protocol [49], NP(Fl) was synthesized by Suzuki coupling reaction using an Fl-based diboronic acid as follows: PBPVS-*b*-PAThrOH (BPVS/AThrOH = 12/88, 35 mg, 0.010 mmol based on the bromide unit), FlB_2_(pin)_2_ (0.0446 g, 0.10 mmol), and Pd(PPh_3_)_4_ (4.3 mg, 0.0037 mmol) were placed in a round bottom flask under a nitrogen atmosphere. After 5 mL of ethanol was added, the resultant mixture was stirred at room temperature for 30 min, until the mixture was completely dissolved. An aqueous solution of Na_2_CO_3_ (20 wt% in water, 0.15 mL) was added, and then the reaction mixture was stirred at 80 °C for 24 h in dark. After the reaction, the product was purified by dialysis with water for 1 day, followed by THF for two days. Finally, the resulting product was dried under vacuum at room temperature to afford NP(Fl). NP(dTh) was prepared using the same synthetic procedure with dThB_2_(pin)_2_ as a cross-linker. The solubilities of NP(dTh) and NP(Fl) are shown in Table S4.

### 2.4. Detection of Amine Compounds

In a vial, 1.0 mL of PPD aqueous solution (5.0 mM) was mixed with 9.0 mL of aqueous NP(Fl) solution (0.1 mg/mL) at room temperature to afford the mixed sample. The fluorescence spectra of the mixed solution are measured with excitation at 340 nm, and the fluorescence intensity at 402 nm is recorded.

### 2.5. Instrumentation

^1^H NMR spectra were collected on a JEOL (Tokyo, Japan) JNM-ECX400 (resonance frequency of 400 MHz). Size-exclusion chromatography (SEC) was performed using a Tosoh HPLC HLC-8220 system equipped with four consecutive hydrophilic vinyl polymer-based gel columns [TSK-GELs (bead size, exclusion limited molecular weight): α-M (13 μm, >1 × 10^7^), α-4000 (10 μm, 4 × 10^5^), α-3000 (7 μm, 9 × 10^4^), α-2500 (7 μm, 5 × 10^3^)] and a guard column [TSK-guardcolumn α]. The system was operated with refractive index and ultraviolet detectors at 40 °C at a flow rate of 1.0 mL/min using DMF containing 10 mM LiBr as the mobile phase. The molecular weights and dispersities were evaluated using polystyrene standards (Tosoh, molecular range 1050–1090000) as a calibration. The circular dichroism (CD) was recorded on a JASCO (Tokyo, Japan) J-720 spectropolarimeter. The UV–vis spectra and fluorescence spectra were recorded on a JASCO (Tokyo, Japan) V-630BIO UV–vis spectrophotometer and a JASCO FP-6100 spectrofluorophotometer, respectively. Fluorescence quantum yields of solutions were determined relative to 9,10-diphenylanthracene (sublimation grade) in cyclohexane [56]. Dynamic light scattering (DLS) was performed using a Zetasizer Nano (Sysmex/Marvern, Kobe, Japan) with a He-Ne laser (633 nm) at a detection angle of 173°. The Z-averaged hydrodynamic diameters were evaluated, as described previously [63]. Zeta potential data in aqueous solutions were collected on a Zetasiser Nano-ZS instrument (Sysmex/Marvern, Kobe, Japan). Transmission electron microscopy (TEM) images were acquired on a JEOL (Tokyo, Japan) TEM-2100F field emission electron microscope at an accelerating voltage of 200 kV. A droplet of the diluted NP solution (3 mg/mL) was directly dropped on carbon-coated Cu grids, followed by drying at room temperature in air.

## 3. Results

### 3.1. Synthesis of Threonine-Containing Amphiphilic Block Copolymer

The synthetic strategy for fluorescent NPs with threonine-based shells is illustrated in Scheme 1. Initially, a dithiocarbamate-terminated hydrophobic PBPVS was prepared by the RAFT polymerization of BPVS, followed by the RAFT polymerization of a threonine-containing AThrOH using the PBPVS macro-CTA (Scheme S1). The polymerization of [AThrOH]_0_/[Macro-CTA]_0_/[AIBN]_0_ = 400 and 200/2/1 afforded amphiphilic block copolymers with different comonomer compositions (BPVS/AThrOH = 12/88 and 24/76, *M*_n,SEC_ = 22,500–23,000, and *M*_w_/*M*_n_ = 1.42–1.49), as confirmed by ^1^H NMR and SEC measurements (Table S3 and Figure S4). The amphiphilic block copolymers were designed using a long hydrophilic PAThrOH block and a sufficiently short cross-linkable PBPVS chain to allow for dissolution in an ethanol/water mixed solvent at a moderate concentration during the core cross-linking reaction. The parent PBPVS-*b*-PAThrOH existed predominantly in the unimolecular form (*D*_h_ < 10 nm) with a small portion of the aggregated form (*D*_h_ > 160 nm) in DMF, as confirmed by DLS (Figure S4c), which is a good solvent for both PAThrOH and PBPVS. In contrast, a micelle-like assembled structure was mainly observed in methanol because of the selective solubility of the PAThrOH shell with an insoluble PBPVS chain in the core.

### 3.2. Synthesis and Fluorescent Properties of Core-Cross-Linked NPs

The fluorescent NP(dTh) and NP(Fl) were prepared by the in situ coupling reaction of the bromine-containing BPVS unit of PBPVS-*b*-PAThrOH with dTh- and Fl-based diboronic acid derivatives, respectively. The Suzuki reaction of PBPVS-*b*-PAThrOH (BPVS/AThrOH = 12/88) was performed in a selective solvent (H_2_O/ethanol = 0.03 vol%) following a previously published method ([Br]/[diboronic acid] = 1/10 in the presence of Pd(PPh_3_)_4_ and Na_2_CO_3_ under reflux for 24 h) [49]. The self-assembly of the threonine-containing block copolymer in the selective solvent provides a hydrophobic environment with the PBPVS segment in the core, which is available for the in situ coupling reaction to afford the targeted fluorescent core-cross-linked NPs in reasonable yields (Table 1). Under these conditions, the hydrophobic Fl-based and dTh-based coupling agents were incorporated into the micelles, leading to site-selective inter- and intra-molecular coupling in the core. The core fragments of the resulting NP(dTh) and NP(Fl) consisted of symmetric diphenyl dTh and Fl units (Scheme 1).

**Figure 1:**
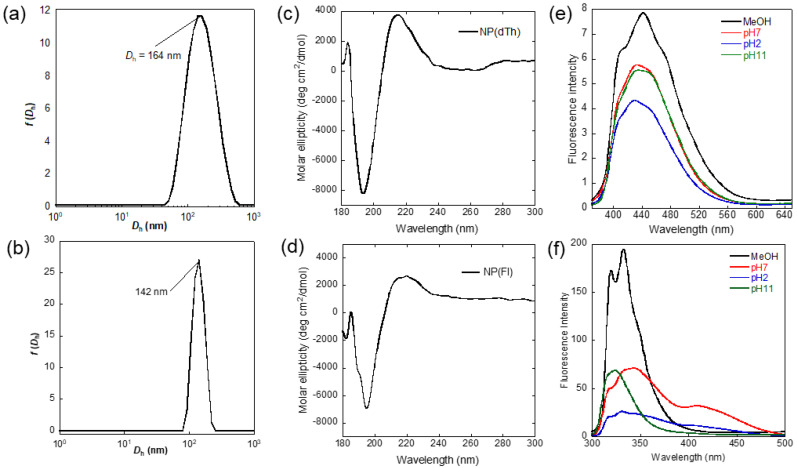
DLS traces of (**a**) NP(dTh) and (**b**) NP(Fl) in CH_3_OH (conc. = 2.0 mg/mL), CD spectra of (**c**) NP(dTh) and (**d**) NP(Fl) in H_2_O (conc. = 0.03 mg/mL), and fluorescence spectra of (**e**) NP(dTh) in CH_3_OH and H_2_O (conc. = 1.0 mg/mL) and (**f**) NP(Fl) in CH_3_OH (conc. = 0.001 mg/mL) and H_2_O (conc. = 0.01 mg/mL).

DLS analysis was conducted to confirm the formation of the core-cross-linked NPs in methanol, which indicated reasonable hydrodynamic diameters (*D*_h_ = 164 nm and 142 nm for NP(dTh) and NP(Fl), respectively), as shown in Figure 1a,b. The uniform DLS traces indicated the presence of spherical core-shell NPs with a PAThrOH shell, which contributed to the enhanced solubility in polar solvents. NP(dTh) and NP(Fl) were uniformly dispersed in methanol (conc. = 2.0 mg/mL) and acted as single, dissolved NPs, which are distinct from the pristine amphiphilic block copolymers (Figure S4c). Similar spherical core-shell NPs with slightly larger particle sizes (*D*_h_ = 220 nm and 190 nm) were observed by DLS in H_2_O at pH = 7 (Figures S5 and S6). The CD spectra exhibited negative and positive Cotton effects at 190 and 215 nm (Figure 1c,d), respectively, implying the presence of a threonine-based chain at the surface of both NPs, independent of the core structure. These results suggest that in situ core cross-linking of the threonine-containing amphiphilic block copolymer in the presence of dTh- and Fl-based coupling agents in the selective solvent proceeded efficiently to afford stable NP(dTh) and NP(Fl) with predetermined core-shell structures with cross-linked cores.

**Figure 2:**
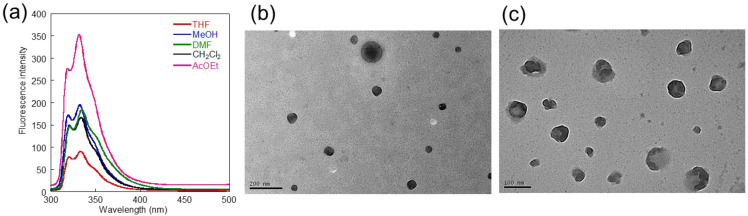
(**a**) Fluorescence spectra of NP(Fl) in different organic solvents (conc. = 0.001 mg/mL), and TEM images of NP(Fl)s prepared using (**b**) ethyl acetate and (**c**) THF solutions.

The optical properties of NP(dTh) and NP(Fl) were investigated using UV–vis and fluorescence spectra in methanol, showing a significantly higher fluorescence intensity of NP(Fl) compared to that of NP(dTh) (Figure 1e,f). NP(Fl) exhibited a maximum absorption peak at 279 nm in methanol (Figure S6), with a strong emission maximum at 333 nm in the fluorescence spectrum. NP(Fl) exhibited a relatively high quantum yield (Φ = 0.22) in methanol, originating from the high fluorescence of the fluorene unit in the core. NP(dTh) exhibited an absorption centered at approximately 340 nm (Figure S5), and the maximum fluorescence was detected at 446 nm. A relatively low Φ (0.04) was observed for NP(dTh) compared to that of NP(Fl), which was probably due to aggregation-induced quenching in methanol. In an aqueous solution at pH = 7, NP(Fl) also showed a high fluorescence intensity (Φ = 0.58), compared to that of NP(dTh) (Φ = 0.16). In contrast, various AIE molecules with similar molecular designs and properties have recently been developed and employed as luminogens, otherwise known as AIEgens, that exhibit emission in the solid state. In addition to the more well-known AIEgens, such as tetraphenylethylene derivatives, fluorene derivatives have been frequently investigated as AIEgens [64,65,66]. In the present system, NP(Fl) with the diphenyl Fl core can be recognized as AIE-active NPs, showing strong emission in the aggregated and the solution state, owing to the restricted rotation between the phenyl-Fl units in the cross-linked core. The influence of solvent polarity on the fluorescence peak position/intensity and quantum yield of NP(Fl) was then investigated (Table 2). The highest quantum yield of 0.72 was obtained in ethyl acetate, and it decreased in the order of 0.31, 0.23, 0.22, and 0.04 in CH_2_Cl_2_, DMF, methanol, and THF, respectively. As illustrated in Figure 2a, the emission wavelength and profile of NP(Fl) were maintained and were independent of the solvent, suggesting no new fluorophore formation. The particle size and distribution of NP(Fl) determined by DLS in DMF and THF were similar to those in methanol (Figure S7b), implying no detectable interparticle aggregation. A similar tendency was observed in AIE-active NPs with fixed tetraphenylethene-based units in the cross-linked cores, which displayed characteristic fluorescence regardless of environmental changes, such as solvent polarities [56,57].

**Figure 3:**
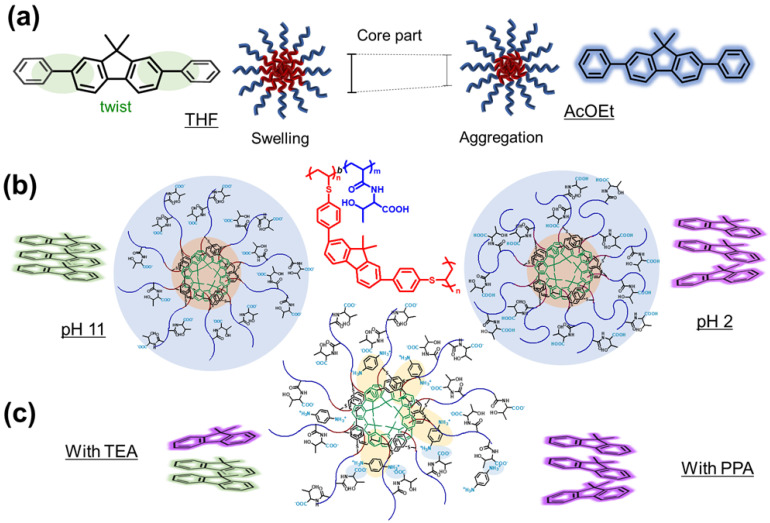
Proposed mechanism of the fluorescence property of NP(Fl) in (**a**) different organic solvents, (**b**) aqueous solutions at different pH values, and (**c**) aqueous solutions in the presence of amine compounds (TEA and PPD).

TEM measurements were then conducted to evaluate the solvent-dependent change in the size and morphology of the cross-linked NP(dTh) and NP(Fl) in the dried state. In both cases, the formation of uniform NPs, which existed predominantly in isolated states without aggregation, was observed (Figure 2b,c and Figure S5a). TEM images of the NP(Fl) samples prepared from different solutions are shown in Figure 2b,c and Figure S8, implying a smaller particle core size in ethyl acetate than that in THF. Aggregated forms were occasionally detected in THF (Figure S8), which comprised many particles. Similar to the AIE system, the rotation between the fluorene and two benzene rings probably weakens the fluorescence in the swollen state, such as in solutions. In contrast, the aggregation of the non-swollen state in ethyl acetate reduced the inactivation processes, such as molecular motion, resulting in enhanced fluorescence intensity (Figure 3a). Therefore, there is sufficient space inside the core to twist and weaken the fluorescence in THF, and such loosely and tightly aggregated states may cause differences in the quantum yield, depending on the solvent polarity.

### 3.3. pH-Dependent Fluorescence and Detection for Amine Compounds

The pH responsiveness of the aqueous NP(Fl) solution was analyzed using DLS, UV–vis, and fluorescence measurements. As shown in Table 1, the particle sizes and zeta potentials determined by DLS were 190 and 220 nm and −47 and −46 mV at a pH of 7 and 11, respectively, which were significantly different from those at pH 2 (size = 122 nm and zeta potential = −2.1 mV). The UV–vis spectra showed additional absorbance at 350 nm at pH 11, and the highest absorbance was observed at pH 11, which may be due to an increase in the conjugate length (Figure S6a). Figure 1f displays the pH-dependent change in the fluorescence peak of NP(Fl). These results indicate that PAThrOH is fully and partially deprotonated at pH 7 and 11, whereas PAThrOH existed in a non-ionized state at pH 2. The difference between the ionized and non-ionized states of the PAThrOH chains on the shell may affect the aggregated and swollen states in the diphenyl Fl core, leading to a pH-dependent color change. 

**Figure 4:**
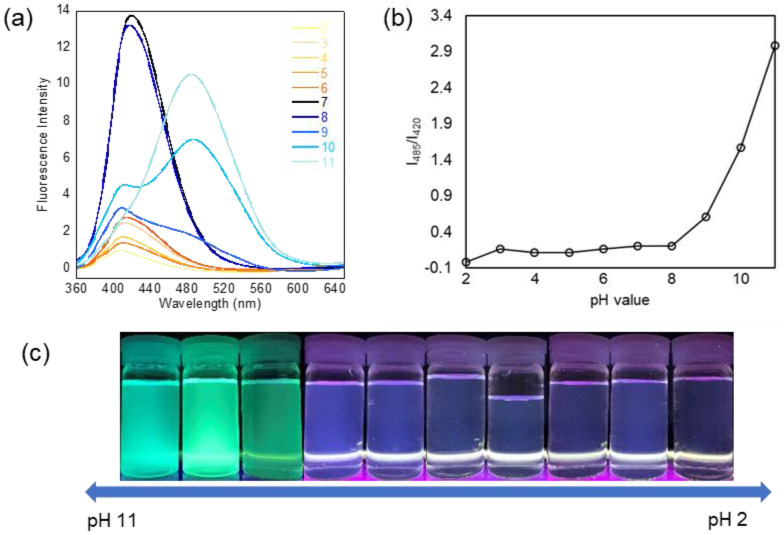
(**a**) Fluorescence spectra in H_2_O at different pH values (conc. = 0.1 mg/mL, λ^abs^ = 340 nm), (**b**) I_4__85_/I_420_ plots, and (**c**) photographs of NP(Fl) at different pH values after irradiation.

The optical and sensing properties of the core-cross-linked NP(Fl), which detects the pH change and presence of amine compounds, were evaluated via fluorescence measurements excited at 340 nm. As shown in Figure 4, two main peaks are clearly visible. The peaks at 420 and 485 nm were due to the fluorene unit and π–π stacking, respectively. Furthermore, the fluorescence intensity of the latter peak increased at pH 10 and 11. Photographs of the aqueous solutions irradiated with a wavelength of 365 nm (UV irradiation) exhibited a color change from blue in the pH range of 2–8 to green at pH 9–11. A plot of the fluorescence intensity ratio between 485 and 420 nm relative to pH (Figure 4b) indicates a switch from the pristine fluorene-based emission to the stacking-derived one under basic conditions, suggesting that the presence of the carboxylate anion in the AThrOH unit may induce π–π stacking (Figure 3b).

The pH responsiveness of the aqueous NP(Fl) solution was also evaluated using CD measurements (Figure S9). The positive and negative Cotton effects observed in the CD spectra of the aqueous NP(Fl) solution at pH 7 and 11 are distinct from those at pH 2. Because PAThrOH is a weak polyelectrolyte with a pH-responsive carboxylic acid, the PAThrOH chain covalently linked to the hydrophobic core with the Fl unit demonstrates pH-dependent switching from the non-ionized to ionized state in water. The presence of the carboxylate anion would cause a strong repulsion between the PAThrOH chains under basic conditions, which may help afford a swollen state in the core, resulting in a change in the emission properties and size of the core-cross-linked NPs.

**Figure 5:**
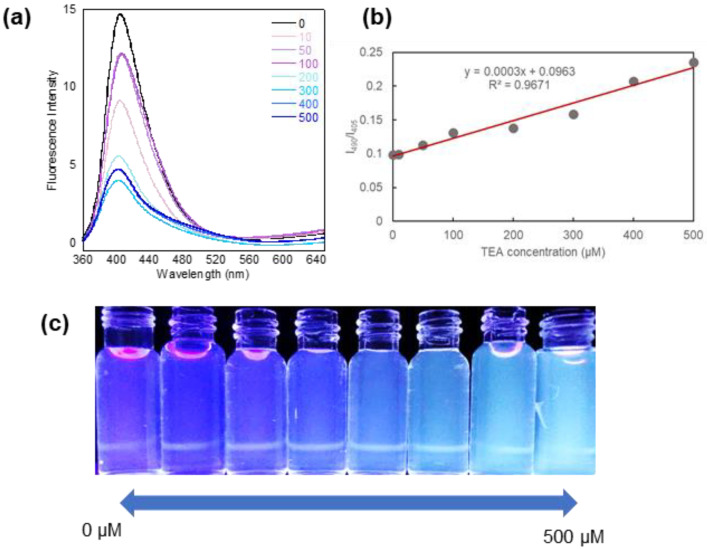
(**a**) Fluorescence spectra in H_2_O (conc. = 0.1 mg/mL, λ^abs^ = 340 nm), (**b**) I_490_/I_405_ plots, and (**c**) photographs of the aqueous solution of NP(Fl) after addition of TEA from 0 to 500 µM.

The pH-responsive color change attributed to the core aggregation of NP(Fl) was then applied to the detection of triethylamine (TEA) and PPD as representative aliphatic and aromatic amine compounds, respectively. The addition of up to 500 µM TEA resulted in a significant decrease in the fluorescence peak intensity at 405 nm, with a negligible increase in the intensity at 490 nm (Figure 5). A plot of the fluorescence intensity between 490 and 405 nm relative to pH indicated a linear change. After UV irradiation, the color of the aqueous solution changed from blue to brighter cyan with the increasing TEA concentration, in which the pH value was changed from 6.8 to 8.7 with an increase in the TEA concentration from 0 to 500 µM. The particle sizes determined by DLS in the aqueous solution (0.1 mg/mL) increased slightly with increasing TEA concentration (Figure S10). The fluorescence behavior in response to the pH change and TEA addition is caused by anion repulsion between the ionized PAThrOH chains. This leads to a change in the aggregated states of the Fl unit in the core of NP(Fl), by which the increased hydrophobic interactions lead to enhanced π–π stacking, resulting in the extension of the conjugation length and change in fluorescence. When TEA was employed, an acid–base interaction additionally contributed to a change in the conformation of the PAThrOH chains, leading to a change in the aggregated and swollen states of the core.

**Figure 6:**
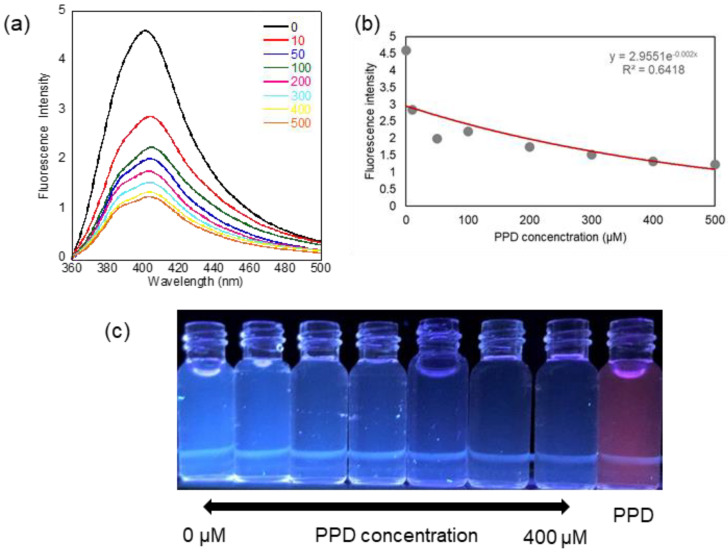
(**a**) Fluorescence spectra in H_2_O (conc. = 0.1 mg/mL, λ^abs^ = 340 nm), (**b**) I_402_ plots, and (**c**) photographs of NP(Fl) after the addition of PPD, with concentration ranging from 0 to 400 µM, and the PPD solution.

In the present system, the NP(Fl) aqueous solution emitted blue fluorescence, and the intensity at 402 nm decreased with the increasing PPD concentration (Figure 6), demonstrating that NP(Fl) acts as a simple turn-off sensor that shows fluorescence quenching after the addition of the target molecule. The addition of 10 µM PPD led to a substantial decrease in the fluorescence intensity, suggesting efficient quenching. A visually detectable difference in the solution color was observed between the reddish-purple PPD solution and the blue NP(Fl)/PPD mixed solution, which became darker with an increase in the PPD concentration. Fluorescence quenching is caused by π–π interactions between Fl in the core of NP(Fl) and the aromatic portion of PPD, which may interfere with the aggregated state of the Fl unit in the cross-linked cores (Figure 3c). Note that a slight color change was observed when using an aliphatic amine (TEA), whereas simple quenching occurred when an aromatic amine (PPD) was used, suggesting the potential of NP(Fl) for the fluorescence sensing of various amine compounds. The fluorescence spectra of the NP(Fl) aqueous solution with PPD excited at 280 nm were also evaluated, where PPD absorbs light more strongly compared to that at 365 nm (Figure S11). As shown in Figure S12, the decrease in the fluorescence intensity at 280 nm with increasing PPD concentration was less significant than that at 365 nm, suggesting that the increasing optical density of the PPD solutions does not interfere with the light-harvesting by NPs and has minimal effect on the fluorescence of the NPs. Although these fluorescent core-cross-linked NP-based sensing systems are still in the preliminary stages and further investigation is required to understand their sensing mechanisms and improve the sensitivity, the molecular design and strategy developed in this study are expected to be applicable to a wide range of polymeric NPs with sensing properties.

## 4. Conclusions

Fluorescent core-cross-linked NP(dTh) and NP(Fl) consisting of symmetric dTh and Fl cores with a weak anionic PAThrOH shell were developed. Targeted NPs were facilely fabricated from a self-assembled block copolymer, PBPVS-*b*-PAThrOH, which was synthesized by RAFT polymerization and subsequent site-selective Suzuki coupling. The DLS, TEM, and CD results show the formation of spherical and stable NPs with uniform sizes and the negative and positive Cotton effects derived from the threonine unit on the shell, which are affected by the solvent polarity and pH range. The cross-linked Fl unit in the core of NP(Fl) provided inherent aggregation states with a hydrophobic environment, resulting in the AIE effect, and the degree of restricted rotation between the fluorene and two benzene rings may contribute to the solvent-dependent fluorescence in organic solvents. In aqueous solution, excellent sensitivity for pH changes originated from the pH-dependent ionized and non-ionized states of the PAThrOH chains, resulting in a change in the emission wavelength and profile owing to different aggregated states with the π–π interaction between the aromatic Fl units in the core. The detection of aliphatic and aromatic amines was mainly attributed to changes in the aggregation states in the cores due to specific interactions between the AThrOH-based shell and amine compounds, resulting in different π–π interactions in the cores and fluorescence quenching with or without slight changes in the emission wavelength and profile for PPD and TEA, respectively. This study demonstrates the potential of fluorescent core-cross-linked NPs with AIE-type tightly condensed cores with a suitable pH-responsive shell and fluorescent core.

## Figures and Tables

**Table 1:** Synthesis and characteristics of NPs.

Product	Yield *^a^*	Solvent	*D*_h_^*b*^ (nm)	Zeta Potential	λ_max_^abs^ ^*c*^	λ_max_^em^ ^*d*^	Φ *^e^*
NP(dTh)	54	CH_3_OH	164	-	340	446	0.04
		H_2_O (pH2)	142	−2.0	341	430	0.14
		H_2_O (pH7)	220	−47.7	341	434	0.16
		H_2_O (pH11)	142	−44.5	349	435	0.04
NP(Fl)	35	CH_3_OH	142	-	279	333	0.22
		H_2_O (pH2)	122	−2.1	267	333	0.06
		H_2_O (pH7)	190	−46.6	274	343	0.58
		H_2_O (pH11)	220	−45.8	267	324	0.03

*^a^* Purification by dialysis in H_2_O for one day and in THF for two days. *^b^* DLS measurements at 25 °C in CH_3_OH and H_2_O (conc. = 2.0 mg/mL). *^c^* Maximum absorption wavelength and *^d^* maximum emission wavelength in CH_3_OH (1.0 mg/mL for NP(dTh) and 0.001 mg/mL for NP(Fl)) and H_2_O (1.0 mg/mL for NP(dTh) and 0.01 mg/mL for NP(Fl)). *^e^* Using 9,10-diphenylanthracene (Φ = 0.95) as a standard.

**Table 2:** Optical property of NP(Fl) in organic solvents.

Solvent	λ_max_^abs^^*a*^	λ_max_^em^^*b*^	Φ *^c^*
THF	276	334	0.04
CH_3_OH	280	333	0.22
DMF	280	335	0.23
CH_2_Cl_2_	290	334	0.31
AcOEt	290	332	0.72

*^a^* Maximum absorption wavelength and *^b^* maximum emission wavelength (conc. = 0.001 mg/mL). *^c^* Using 9,10-diphenylanthracene (Φ = 0.95) as a standard.

## Data Availability

The data presented in this study are available on request from the corresponding author.

## Supplementary Materials

The following supporting information can be downloaded at: https://www.mdpi.com/article/10.3390/polym14071362/s1, Scheme S1: Synthesis of PBPVS; Scheme S2: Synthesis of PBPVS-*b*-PAThrOH; Figure S1: 1H NMR spectrum of PBPVS in CDCl3; Figure S2: SEC curves of PBPVS prepared by RAFT polymerization at different [M]/[CTA] ratios; Figure S3: SEC curves of PBPVS prepared for different times; Figure S4: (a) 1H NMR spectrum in DMSO-d6, (b) SEC curves, and (c) DLS traces of PBPVS-b-PAThrOH; Figure S5: (a) TEM image, (b) DLS traces, (c) UV–vis spectra, and (d) CD spectra of NP(dTh) in MeOH and water at different pH values; Figure S6: (a) UV–vis spectra and (b) DLS traces of NP(Fl) in MeOH and water; Figure S7: (a) UV–vis spectra of NP(Fl) in different organic solvents (conc. = 0.001 mg/mL) and (b) DLS traces in different solvents (2.0 mg/mL); Figure S8: TEM images of NP(Fl) samples prepared from diluted (a,b) AcOEt, (c,d) DMF, (e,f) CHCl3, and (g,h) THF solutions; Figure S9: (a) DLS traces, (b) UV–vis spectra, and (c) CD spectra of NP(Fl) in H2O at different pH values; Figure S10: (a) UV–vis spectra, (b) I450 plots, (c) DLS traces, and (d) diameter plots of NP(Fl) in H2O after addition of TEA; Figure S11: UV–vis spectra of NP(Fl) in H2O after addition of PPD; Figure S12: Fluorescence spectra of NP(Fl) in H2O (conc. = 0.1 mg/mL, λabs = 280 nm) after addition of PPD; Table S1: Synthesis of PBPVS in bulk at different [M]/[CTA] ratios and 60 °C for 24 h; Table S2: Synthesis of PBPVS in bulk at 60 °C; Table S3: Synthesis of PBPVS-b-PAThrOH in DMF at 60 °C for 24h; Table S4: Solubility of block copolymer, NP(dTh), and NP(Fl); Table S5: Summary of fluorescence quantum yields of NP(Fl) in H2O after addition of PPD.

## References

[1] Pecher J., Mecking S. (2010). Nanoparticles of Conjugated Polymers. Chem. Rev..

[2] Grigalevicius S., Forster M., Ellinger S., Landfester K., Scherf U. (2006). Excitation energy transfer from semi-conducting polymer nanoparticles to surface-bound fluorescent dyes. Macromol. Rapid Commun..

[3] Wu C., Peng H., Jiang Y., McNeill J. (2006). Energy transfer mediated fluorescence from blended conjugated polymer nanoparticles. J. Phys. Chem. B.

[4] Wu C., Szymanski C., McNeill J. (2006). Preparation and encapsulation of highly fluorescent conjugated polymer nanoparticles. Langmuir.

[5] Li K., Liu B. (2014). Polymer-encapsulated organic nanoparticles for fluorescence and photoacoustic imaging. Chem. Soc. Rev..

[6] Tuncel D., Demir H.V. (2010). Conjugated polymer nanoparticles. Nanoscale.

[7] Kietzke T., Neher D., Landfester K., Montenegro R., Guntner R., Scherf U. (2003). Novel approaches to polymer blends based on polymer nanoparticles. Nat. Mater..

[8] Fisslthaler E., Bluemel A., Landfester K., Scherf U., List E.J.W. (2008). Printing functional nanostructures: A novel route towards nanostructuring of organic electronic devices via soft embossing, inkjet printing and colloidal self assembly of semiconducting polymer nanospheres. Soft Matter.

[9] Mauthner G., Landfester K., Köck A., Brückl H., Kast M., Stepper C., List E.J.W. (2008). Inkjet printed surface cell light-emitting devices from a water-based polymer dispersion. Org. Electron..

[10] Wu C., Szymanski C., Cain Z., McNeill J. (2007). Conjugated Polymer Dots for Multiphoton Fluorescence Imaging. J. Am. Chem. Soc..

[11] Tian Z., Wu W., Wan W., Li A.D.Q. (2009). Single-Chromophore-Based Photoswitchable Nanoparticles Enable Dual-Alternating-Color Fluorescence for Unambiguous Live Cell Imaging. J. Am. Chem. Soc..

[12] Zhu L., Wu W., Zhu M.-Q., Han J.J., Hurst J.K., Li A.D.Q. (2007). Reversibly photoswitchable dual-color fluorescent nanoparticles as new tools for live-cell imaging. J. Am. Chem. Soc..

[13] Moon J.H., McDaniel W., MacLean P., Hancock L.F. (2007). Live-cell-permeable poly(p-phenylene ethynylene). Angew. Chem. Int. Ed..

[14] Zhu M.-Q., Zhu L., Han J.J., Wu W., Hurst J.K., Li A.D.Q. (2006). Spiropyran-Based Photochromic Polymer Nanoparticles with Optically Switchable Luminescence. J. Am. Chem. Soc..

[15] Fischer C.S., Baier M.C., Mecking S. (2013). Enhanced Brightness Emission-Tuned Nanoparticles from Heterodifunctional Polyfluorene Building Blocks. J. Am. Chem. Soc..

[16] Chen C.-P., Huang Y.-C., Liou S.-Y., Wu P.-J., Kuo S.-Y., Chan Y.-H. (2014). Near-Infrared Fluorescent Semiconducting Polymer Dots with High Brightness and Pronounced Effect of Positioning Alkyl Chains on the Comonomers. ACS Appl. Mater. Interfaces.

[17] Yang C., Liu H., Zhang Y., Xu Z., Wang X., Cao B., Wang M. (2016). Hydrophobic-Sheath Segregated Macromolecular Fluorophores: Colloidal Nanoparticles of Polycaprolactone-Grafted Conjugated Polymers with Bright Far-Red/Near-Infrared Emission for Biological Imaging. Biomacromolecules.

[18] Yang C., Huang S., Wang X., Wang M. (2016). Theranostic unimolecular micelles of highly fluorescent conjugated polymer bottlebrushes for far red/near infrared bioimaging and efficient anticancer drug delivery. Polym. Chem..

[19] Wang Y., Zhou K., Huang G., Hensley C., Huang X., Ma X., Zhao T., Sumer B.D., DeBerardinis R.J., Gao J. (2014). A nanoparticle-based strategy for the imaging of a broad range of tumours by nonlinear amplification of microenvironment signals. Nat. Mater..

[20] Ng K.K., Zheng G. (2015). Molecular Interactions in Organic Nanoparticles for Phototheranostic Applications. Chem. Rev..

[21] Canfarotta F., Whitcombe M.J., Piletsky S.A. (2013). Polymeric nanoparticles for optical sensing. Biotechnol. Adv..

[22] Li C., Liu S. (2012). Polymeric assemblies and nanoparticles with stimuli-responsive fluorescence emission characteristics. Chem. Commun..

[23] Chen M., Yin M. (2014). Design and development of fluorescent nanostructures for bioimaging. Prog. Polym. Sci..

[24] Hasseb A.A., Ghani N.d.T.A., Shehab O.R., El Nashar R.M. (2022). Application of molecularly imprinted polymers for electrochemical detection of some important biomedical markers and pathogens. Curr. Opin. Electrochem..

[25] Ziai Y., Petronella F., Rinoldi C., Nakielski P., Zakrzewska A., Kowalewski T.A., Augustyniak W., Li X., Calogero A., Sabala I. (2022). Chameleon-inspired multifunctional plasmonic nanoplatforms for biosensing applications. NPG Asian Mater..

[26] Schaeferling M. (2012). The Art of Fluorescence Imaging with Chemical Sensors. Angew. Chem. Int. Ed..

[27] Wu J., Liu W., Ge J., Zhang H., Wang P. (2011). New sensing mechanisms for design of fluorescent chemosensors emerging in recent years. Chem. Soc. Rev..

[28] Yuan L., Lin W., Zheng K., He L., Huang W. (2013). Far-red to near infrared analyte-responsive fluorescent probes based on organic fluorophore platforms for fluorescence imaging. Chem. Soc. Rev..

[29] Sathiskumar U., Easwaramoorthi S. (2019). Red-Emitting Ratiometric Fluorescence Chemodosimeter for the Discriminative Detection of Aromatic and Aliphatic Amines. ChemistrySelect.

[30] Mallick A., El-Zohry A.M., Shekhah O., Yin J., Jia J., Aggarwal H., Emwas A.-H., Mohammed O.F., Eddaoudi M. (2019). Unprecedented Ultralow Detection Limit of Amines using a Thiadiazole-Functionalized Zr(IV)-Based Metal-Organic Framework. J. Am. Chem. Soc..

[31] Oliveri I.P., Di Bella S. (2011). Sensitive Fluorescent Detection and Lewis Basicity of Aliphatic Amines. J. Phys. Chem. A.

[32] Hayashi T., Nonoguchi M., Aya T., Ogoshi H. (1997). Molecular recognition of alpha, omega-diamines by metalloporphyrin dimer. Tetrahedron Lett..

[33] Fu Y., Yao J., Xu W., Fan T., He Q., Zhu D., Cao H., Cheng J. (2015). Reversible and “fingerprint” fluorescence differentiation of organic amine vapours using a single conjugated polymer probe. Polym. Chem..

[34] Gao M., Li S., Lin Y., Geng Y., Ling X., Wang L., Qin A., Tang B.Z. (2016). Fluorescent Light-Up Detection of Amine Vapors Based on Aggregation-Induced Emission. ACS Sens..

[35] Gao L.-f., Lin X., Hai X., Chen X.-w., Wang J.-h. (2018). Polymeric Ionic Liquid-Based Fluorescent Amphiphilic Block Copolymer Micelle for Selective and Sensitive Detection of p-Phenylenediamine. ACS Appl. Mater. Interfaces.

[36] Gu M., Duan J., Mao Q., Zhang S., Lv J. (2019). Direct chemiluminescent sensing of para-Phenylenediamine over its isomers and analogues via luminol diazotization. Sens. Actuators B Chem..

[37] Zhao Y.-J., Miao K., Zhu Z., Fan L.-J. (2017). Fluorescence Quenching of a Conjugated Polymer by Synergistic Amine-Carboxylic Acid and pi-pi Interactions for Selective Detection of Aromatic Amines in Aqueous Solution. ACS Sens..

[38] Rheingans O., Hugenberg N., Harris J.R., Fischer K., Maskos M. (2000). Nanoparticles Built of Cross-Linked Heterotelechelic, Amphiphilic Poly(dimethylsiloxane)-b-poly(ethylene oxide) Diblock Copolymers. Macromolecules.

[39] Henselwood F., Liu G. (1997). Water-soluble nanospheres of poly(2-cinnamoylethyl methacrylate)-block-poly(acrylic acid). Macromolecules.

[40] Guo A., Liu G., Tao J. (1996). Star Polymers and Nanospheres from Cross-Linkable Diblock Copolymers. Macromolecules.

[41] Chen D., Peng H., Jiang M. (2003). A Novel One-Step Approach to Core-Stabilized Nanoparticles at High Solid Contents. Macromolecules.

[42] Bronich T.K., Keifer P.A., Shlyakhtenko L.S., Kabanov A.V. (2005). Polymer Micelle with Cross-Linked Ionic Core. J. Am. Chem. Soc..

[43] Huang H., Hoogenboom R., Leenen M.A.M., Guillet P., Jonas A.M., Schubert U.S., Gohy J.-F. (2006). Solvent-Induced Morphological Transition in Core-Cross-Linked Block Copolymer Micelles. J. Am. Chem. Soc..

[44] Iijima M., Nagasaki Y., Okada T., Kato M., Kataoka K. (1999). Core-Polymerized Reactive Micelles from Heterotelechelic Amphiphilic Block Copolymers. Macromolecules.

[45] Pioge S., Nesterenko A., Brotons G., Pascual S., Fontaine L., Gaillard C., Nicol E. (2011). Core Cross-Linking of Dynamic Diblock Copolymer Micelles: Quantitative Study of Photopolymerization Efficiency and Micelle Structure. Macromolecules.

[46] Anger C., Deubel F., Salzinger S., Stohrer J., Halbach T., Jordan R., Veinot J.G.C., Rieger B. (2013). Organic-Inorganic Hybrid Nanoparticles via Photoinduced Micellation and Siloxane Core Cross-Linking of Stimuli-Responsive Copolymers. ACS Macro Lett..

[47] Nakabayashi K., Oya H., Mori H. (2012). Cross-linked core-shell nanoparticles based on amphiphilic block copolymers by RAFT polymerization and palladium-catalyzed suzuki coupling reaction. Macromolecules.

[48] Mori H., Takano K., Endo T. (2009). RAFT Polymerization of vinylthiophene derivatives and synthesis of block copolymers having cross-linkable segments. Macromolecules.

[49] Abiko Y., Matsumura A., Nakabayashi K., Mori H. (2014). Thermoresponsive core–shell nanoparticles with cross-linked π-conjugate core based on amphiphilic block copolymers by RAFT polymerization and palladium-catalyzed coupling reactions. Polymer.

[50] Nakabayashi K., Noda D., Watanabe Y., Mori H. (2015). Rylene bisimide-based nanoparticles with cross-linked core and thermoresponsive shell using poly(vinyl amine)-based block copolymers. Polymer.

[51] Nakabayashi K., Noda D., Takahashi T., Mori H. (2016). Design of stimuli-responsive nanoparticles with optoelectronic cores by post-assembly cross-linking and self-assembly of functionalized block copolymers. Polymer.

[52] Rudolph T., Schacher F.H. (2016). Selective crosslinking or addressing of individual domains within block copolymer nanostructures. Eur. Polym. J..

[53] Lo C.-T., Watanabe Y., Oya H., Nakabayashi K., Mori H., Chen W.-C. (2016). Non-volatile transistor memory devices using charge storage cross-linked core-shell nanoparticles. Chem. Commun..

[54] Nakabayashi K., Takahashi T., Sugawara R., Lo C.-T., Mori H. (2018). Benzothiadiazole-based donor-acceptor nanoparticles with solvatochromic and thermoresponsive properties. React. Funct. Polym..

[55] Lo C.-T., Watanabe Y., Murakami D., Shih C.-C., Nakabayashi K., Mori H., Chen W.-C. (2019). Donor-Acceptor Core-Shell Nanoparticles and Their Application in Non-Volatile Transistor Memory Devices. Macromol. Rapid Commun..

[56] Furukawa M., Nakabayashi K., Mori H. (2021). Aggregation-induced multicolor luminescent nanoparticles with adaptive and fixed cores derived from brominated tetraphenylethene-containing block copolymer. J. Polym. Sci..

[57] Nakabayashi K., Takata M., Furukawa M., Mori H. (2020). Luminescent core-shell nanoparticles with crosslinked aggregation-induced emission core structures: Emission both in solution and solid states. J. Polym. Sci..

[58] Zhao Y., Higashihara T., Sugiyama K., Hirao A. (2005). Synthesis of functionalized asymmetric star polymers containing conductive polyacetylene segments by living anionic polymerization. J. Am. Chem. Soc..

[59] Nakabayashi K., Abiko Y., Mori H. (2013). RAFT Polymerization of S-vinyl sulfide derivatives and synthesis of block copolymers having two distinct optoelectronic functionalities. Macromolecules.

[60] Shoji K., Nakayama M., Koseki T., Nakabayashi K., Mori H. (2016). Threonine-based chiral homopolymers with multi-stimuli-responsive property by RAFT polymerization. Polymer.

[61] Chiefari J., Mayadunne R.T.A., Moad C.L., Moad G., Rizzardo E., Postma A., Skidmore M.A., Thang S.H. (2003). Thiocarbonylthio compounds (S=C(Z)S-R) in free radical polymerization with reversible addition-fragmentation chain transfer (RAFT polymerization). Effect of the activating group Z. Macromolecules.

[62] Mori H., Nakano S., Endo T. (2005). Controlled Synthesis of Poly(N-ethyl-3-vinylcarbazole) and Block Copolymers via RAFT Polymerization. Macromolecules.

[63] Kanto R., Qiao Y., Masuko K., Furusawa H., Yano S., Nakabayashi K., Mori H. (2019). Synthesis, Assembled Structures, and DNA Complexation of Thermoresponsive Lysine-Based Zwitterionic and Cationic Block Copolymers. Langmuir.

[64] Feng X.J., Peng J., Xu Z., Fang R., Zhang H.-r., Xu X., Li L., Gao J., Wong M.S. (2015). AIE-Active Fluorene Derivatives for Solution-Processable Nondoped Blue Organic Light-Emitting Devices (OLEDs). ACS Appl. Mater. Interfaces.

[65] Naeem K.C., Neenu K., Nair V.C. (2017). Effect of Differential Self-Assembly on Mechanochromic Luminescence of Fluorene-Benzothiadiazole-Based Fluorophores. ACS Omega.

[66] Zhang F., Zhang R., Liang X., Guo K., Han Z., Lu X., Xie J., Li J., Li D., Tian X. (2018). 1, 3-Indanedione functionalized fluorene luminophores: Negative solvatochromism, nanostructure-morphology determined AIE and mechanoresponsive luminescence turn-on. Dye. Pigment..

