# Supplementary material for: Threonine-Based Stimuli-Responsive Nanoparticles with Aggregation-Induced Emission-Type Fixed Cores for Detection of Amines in Aqueous Solutions
Source: Polymers (Basel). 2022 Mar 27;14(7):1362. doi: 10.3390/polym14071362 (PMC9002686; doi:10.3390/polym14071362)
Supplement: Supplementary file 1 [file polymers-14-01362-s001.zip › polymers-1645308-supplementary.pdf]

Supporting Information

Threonine-based stimuli-responsive nanoparticles with aggregation-induced emission-type fixed cores for detection of amines in aqueous solutions

Keita Kataoka, [a] *Kazuhiro Nakabayashi,* [a] *Chen-Tsyr Lo,* [a,b] *and Hideharu Mori* [a*]

[a]Graduate School of Organic Materials Science, Yamagata University, 4-3-16 Jonan, Yonezawa 992-8510 Japan

[b]Department of Materials and Optoelectronic Science, National Sun Yat-Sen University, MS3014,70 Lienhai Road,Kaohsiung 80424 Taiwan

[*] To whom correspondence should be addressed. e-mail: h.mori@yz.yamagata-u.ac.jp

Phone:+81-238-26-3765, Fax: +81-238-26-3092

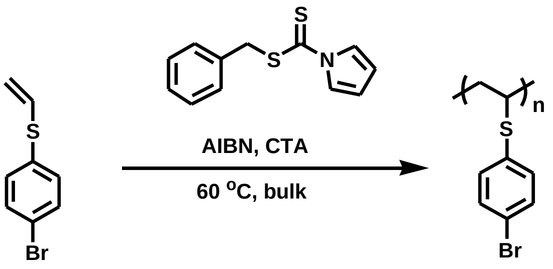

Scheme S1. Synthesis of PBPVS.

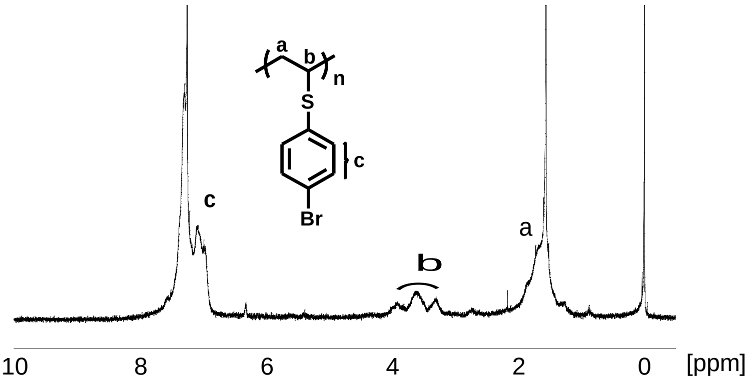

Figure S1. ^{1}H NMR spectrum of PBPVS in $CDCl_3$.

Table S1. Synthesis of PBPVS in bulk at different [M]/[CTA] ratios and 60 °C for 24 h [a)]

Entry	$[Monomer]_0/[CTA]_0$	Conv.[b)] (%)	Yield [c)] (%)	M_n [d)] (theory)	M_n [b)] (^{1}H NMR)	M_n [e)] (SEC)	M_w/M_n [e)] (SEC)
1	25	36	6	2200	3000	1400	1.41
2	50	55	24	6100	5400	2000	1.39
3	100	51	27	11200	10500	3200	1.33

[a)] $[CTA]_0/[AIBN]_0 = 2$. [b)] Calculated by ^{1}H NMR. [c)] Hexane-insoluble part. [d)] The theoretical molecular weight ($M_{n,theory}$) = (MW of BPVS) × $[BPVS]_0/[CTA]_0$ × conv. + (MW of CTA). [e)] Measured by SEC using polystyrene standard in DMF (10 mM LiBr).

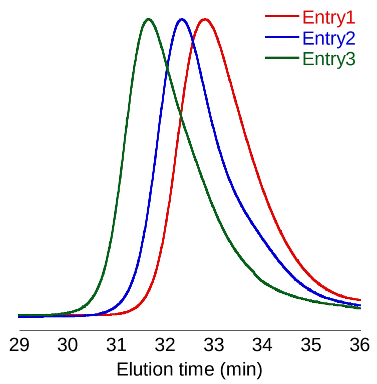

Figure S2. SEC curves of PBPVS prepared by RAFT polymerization at different [M]/[CTA] ratios.

Table S2. Synthesis of PBPVS in bulk at 60 °C [a)]

Entry	Time (h)	Conv. [b)] (%)	Yield [c)] (%)	M_n [d)] (theory)	M_n [b)] (^{1}H NMR)	M_n [e)] (SEC)	M_w/M_n [e)] (SEC)
1	6	19	6	4320	4000	1900	1.32
2	12	41	18	9000	6400	2700	1.34
3	24	51	27	11200	10500	3200	1.33

[a)] $[M]_0/[CTA]_0/[AIBN] = 200/2/1$. [b)] Calculated by ^{1}H NMR. [c)] Hexane-insoluble part. [d)] The theoretical molecular weight ($M_{n,theory}$) = (MW of BPVS) × $[BPVS]_0/[CTA]_0$ × conv. + (MW of CTA). [e)] Measured by SEC using polystyrene standard in DMF (10 mM LiBr).

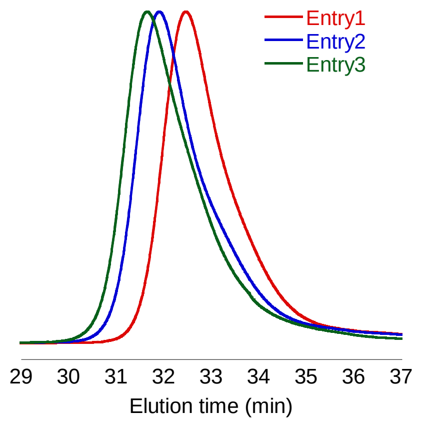

Figure S3. SEC curves of PBPVS prepared for different polymerization times.

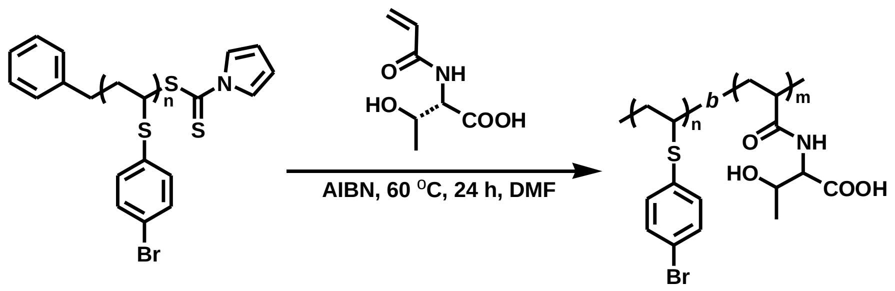

Scheme S2. Synthesis of PBPVS-*b*-PAThrOH.

Table S3. Synthesis of PBPVS-*b*-PAThrOH in DMF at 60 °C for 24h

Entry	$[I]_0$/[Macro-CTA$]_0$/ [Monomer$]_0$	Conv. [a] (%)	Yield [b] (%)	M_n [c] (theory)	M_n [a] (^{1}H NMR)	M_n [d] (SEC)	M_w/M_n [d] (SEC)	n/m [a]
1 [e]	1/2/200	89	54	20800	10700	23000	1.42	24/76
2 [f]	1/2/400	65	54	26000	17000	22500	1.49	12/88

[a] Calculated by ^{1}H NMR. [b] Ethyl acetate insoluble part. [c] The theoretical molecular weight ($M_{n,theory}$) = (MW of AThrOMe) × $[AThrOH]_0/[CTA]_0$ × conv. + (M_n of macro-CTA). [d] Mathylated PBPVS-*b*-PAThrOH was measured by SEC using polystyrene standard in DMF (10 mM LiBr). [e] Macro-CTA (Entry 1) : M_n(SEC) = 2000, M_n(^{1}H NMR) = 5400, M_w/M_n = 1.39. [f] Macro-CTA (Entry 2) : M_n(SEC) = 1700, M_n(^{1}H NMR) = 3500, M_w/M_n = 1.37.

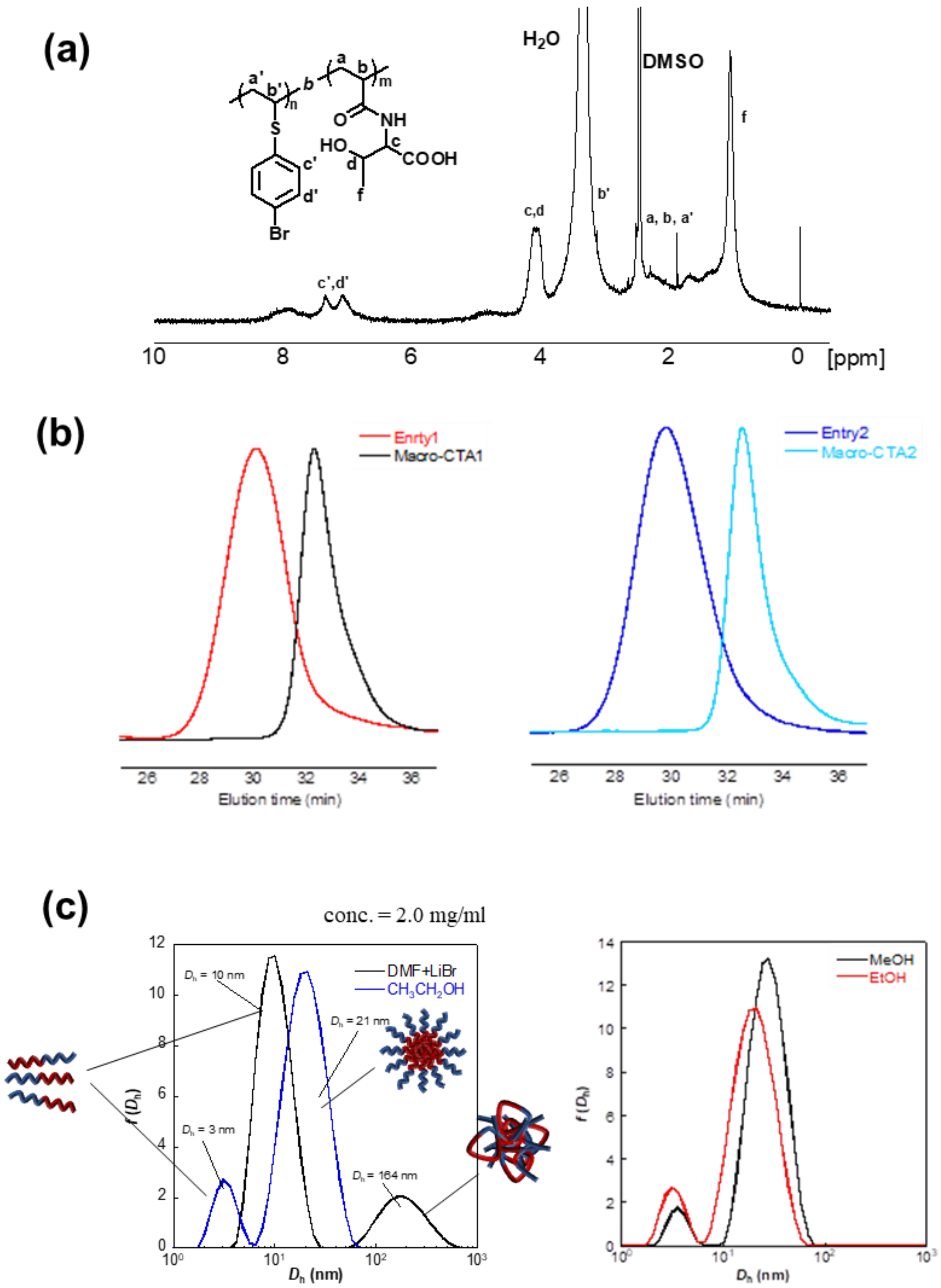

Figure S4. (a) ^{1}H NMR spectrum in DMSO-d_6, (b) SEC curves, and (c) DLS traces of PBPVS-*b*-PAThrOH.

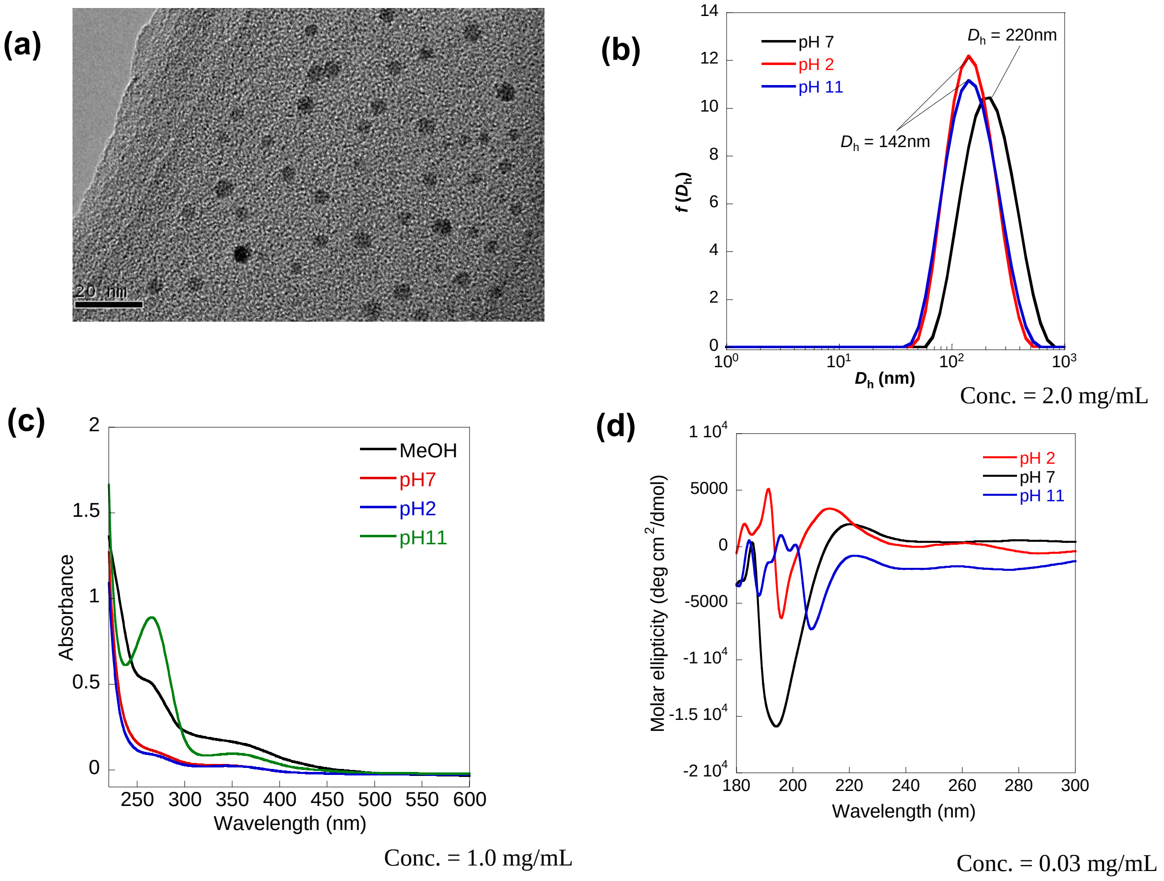

Figure S5. (a) TEM image, (b) DLS traces, (c) UV-vis spectra, and (d) CD spectra of NP(dTh) in MeOH and water at different pH values.

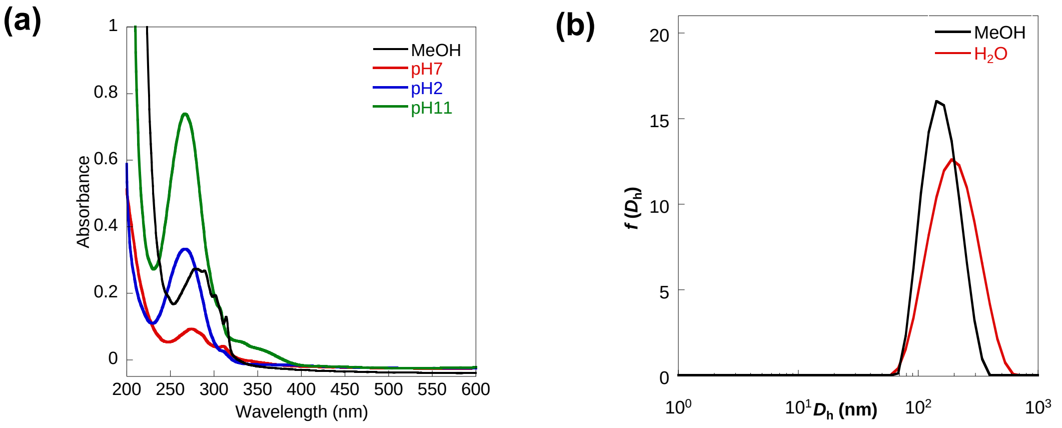

Conc.= 0.001 mg/mL in MeOH
0.01 mg/mL in H_2O (pH2,7,11)

Conc. = 2.0 mg/mL

Figure S6. (a) UV-vis spectra and (b) DLS traces of NP(Fl) in MeOH and water.

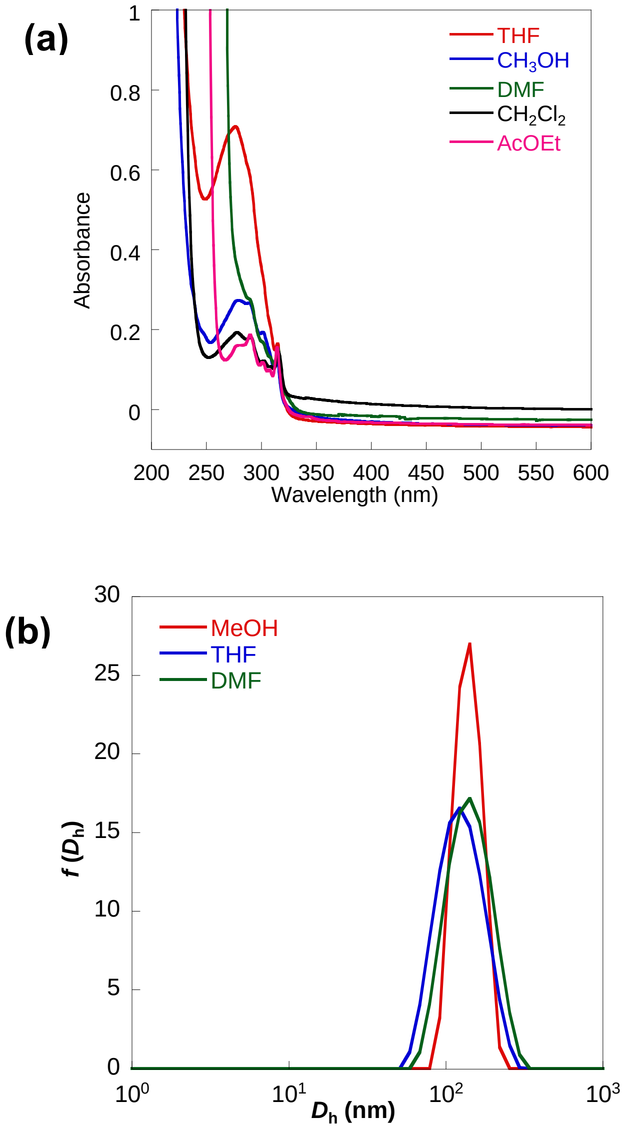

Figure S7. (a) UV-vis spectra of NP(Fl) in different organic solvents (conc. = 0.001 mg/mL) and (b) DLS traces in different solvents (2.0 mg/mL).

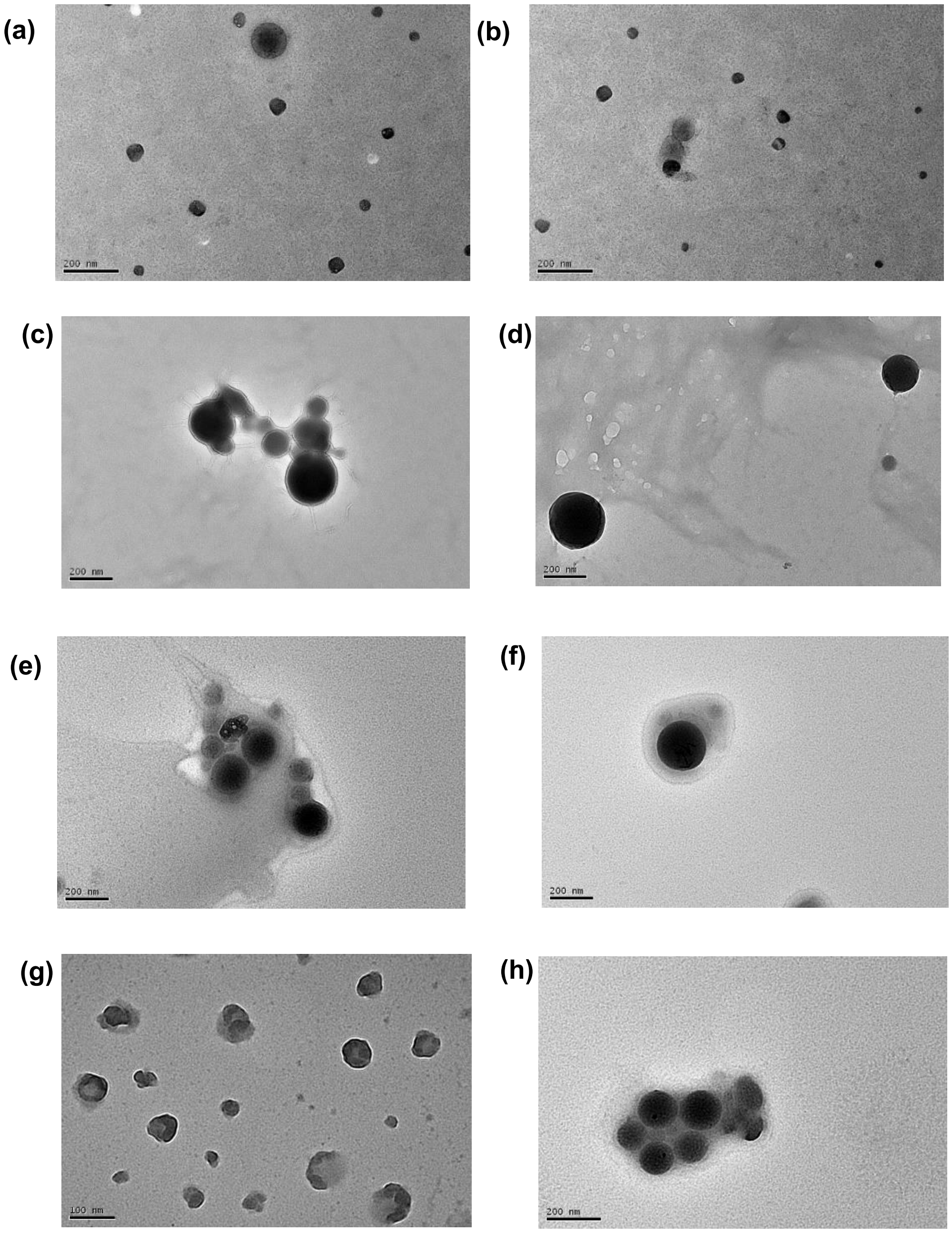

Figure S8. TEM images of NP(Fl) samples prepared from diluted (a, b) AcOEt, (c, d) DMF, (e, f) $CHCl_3$, and (g, h) THF solutions.

Table S4. Solubility of block copolymer, NP(dTh), and NP(Fl)

sample	$CHCl_3$	CH_2Cl_2	THF	CH_3OH	Ethanol
PBPVS-*b*-PAThrOH	−	−	−	+	+
NP(dTh)	+−	+	+	+−	+−
NP(Fl)	+	+	+−	+−	+−

sample	Diethyl ether	Ethyl acetate	1,4-dioxane	hexane	Toluene
PBPVS-b-PAThrOH	−	−	−	−	+−
NP(dTh)	+−	+−	+−	+−	+
NP(Fl)	−	+−	+−	−	+

sample	Acetone	2-propanol	DMF	DMSO	H_2O
PBPVS-*b*-PAThrOH	−	+−	+	+	+
NP(dTh)	+−	+−	+−	+−	+−
NP(Fl)	−	+	+−	+	+−

＋ : Soluble at room temperature, ＋－: partially soluble and/or soluble under diluted conditions, －: Insoluble at room temperature.

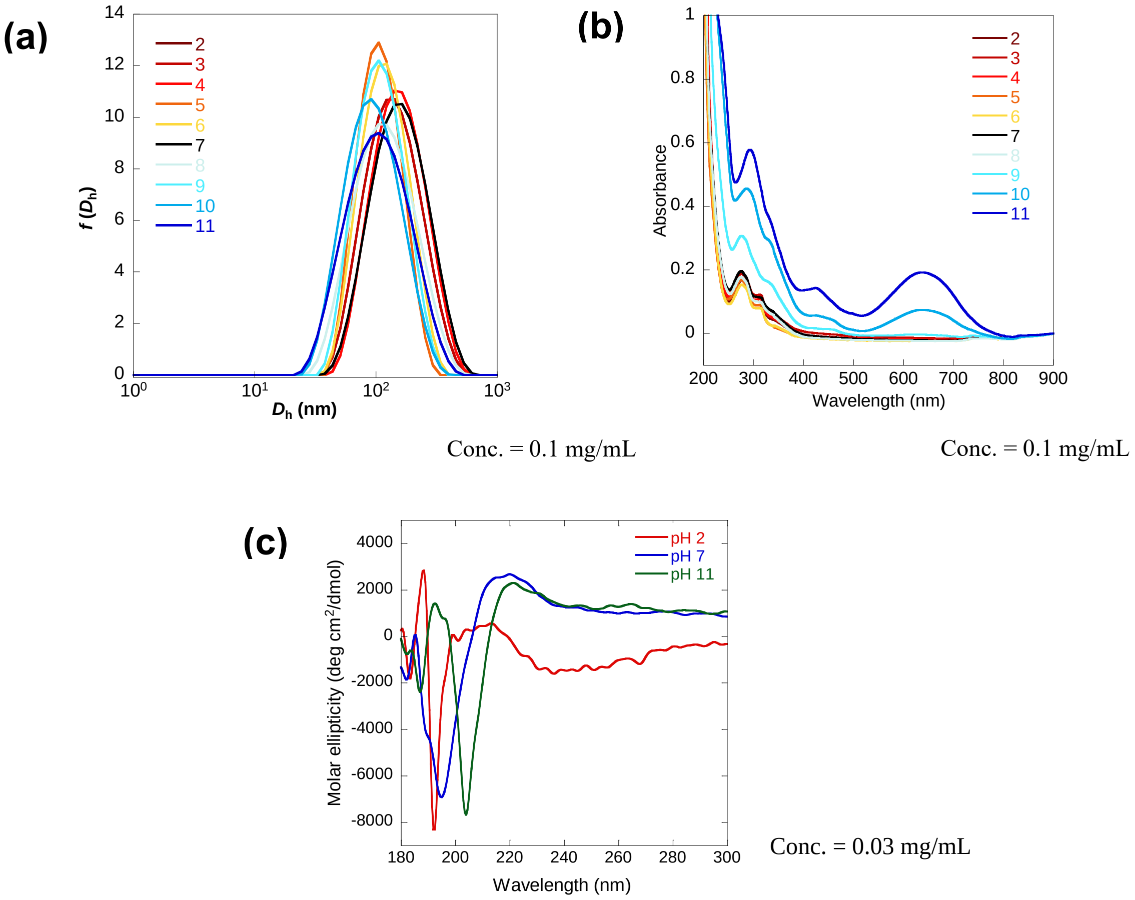

Figure S9. (a) DLS traces, (b) UV-vis spectra, and (c) CD spectra of NP(Fl) in H_2O at different pH values.

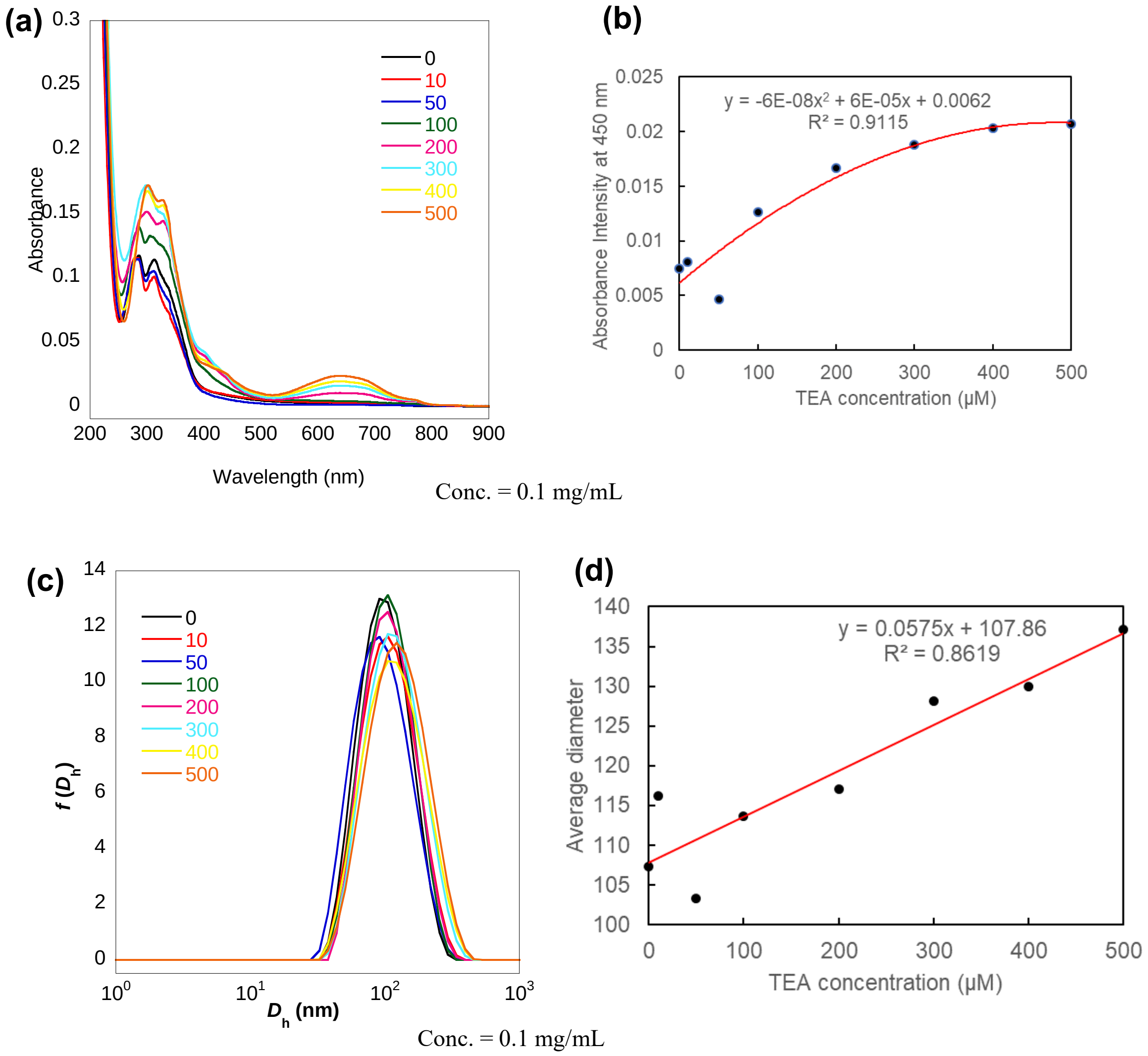

Figure S10. (a) UV-vis spectra, (b) I_{450} plots, (c) DLS traces, and (d) diameter plots of NP(Fl) in H_2O after addition of TEA.

Table S5. Summary of fluorescence quantum yields of NP(Fl) in H_2O after addition of PPD

PPD (μM)	0	10	50	100	200	300	400	500
**Φ**	0.0102	0.00671	0.00423	0.00491	0.00324	0.00275	0.00228	0.00171

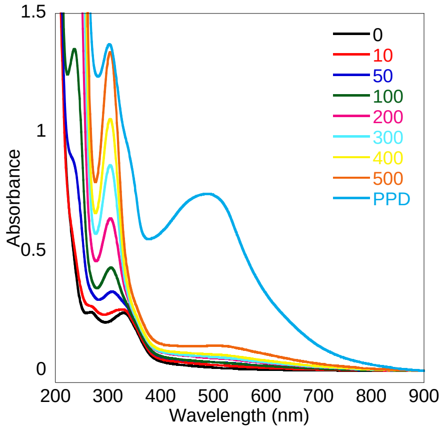

Figure S11. UV-vis spectra of NP(Fl) in H_2O after addition of PPD.

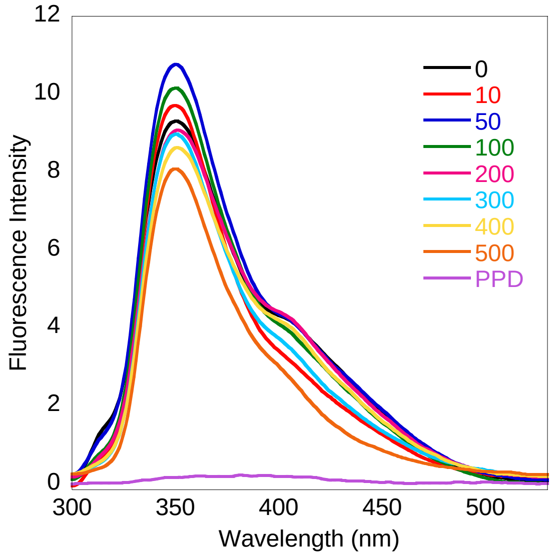

Figure S12. Fluorescence spectra of NP(Fl) in H_2O (conc.= 0.1 mg/mL, λ^{abs} = 280 nm) after addition of PPD.
